# Wearable sensors-based assistive technologies for patient health monitoring

**DOI:** 10.3389/fbioe.2025.1437877

**Published:** 2025-06-02

**Authors:** Nouf Abdullah Almujally, Danyal Khan, Naif Al Mudawi, Mohammed Alonazi, Haifa F. Alhasson, Ahmad Jalal, Hui Liu

**Affiliations:** ^1^ Department of Information Systems, College of Computer and Information Sciences, Princess Nourah bint Abdulrahman University, Riyadh, Saudi Arabia; ^2^ Faculty of Computer Science and AI, Air University, Islamabad, Pakistan; ^3^ Department of Computer Science, National University of Modern Languages NUML, Islamabad, Pakistan; ^4^ Department of Computer Science, College of Computer Science and Information System, Najran University, Najran, Saudi Arabia; ^5^ Department of Information Systems, College of Computer Engineering and Sciences, Prince Sattam bin Abdulaziz University, Al-Kharj, Saudi Arabia; ^6^ Department of Information Technology, College of Computer, Qassim University, Buraydah, Saudi Arabia; ^7^ Department of Computer Science and Engineering, College of Informatics, Korea University, Seoul, Republic of Korea; ^8^ Guodian Nanjing Automation Co., Ltd., Nanjing, China; ^9^ Jiangsu Key Laboratory of Intelligent Medical Image Computing, School of Future Technology, Nanjing University of Information Science and Technology, Nanjing, China; ^10^ Cognitive Systems Lab, University of Bremen, Bremen, Germany

**Keywords:** patient monitoring, wearable sensors, accelerometers, biosensors, healthcare, human-machine interaction, machine learning, deep learning

## Abstract

**Introduction:**

With the advancement of handheld devices, patient health monitoring using wearable devices plays a vital role in overall health monitoring.

**Methods:**

In this article, we have integrated multi-model bio-signals to monitor patient health data during daily life activities continuously. Two well-known datasets from ScientISST MOVE and mHealth have been analyzed. The purpose of this study is to explore the possibilities of using advanced bio-signals for monitoring patient vital signs during daily life activities and predicting favorable and more accurate health-related solutions based on current body health-related real-time measurements.

**Results:**

With the help of machine learning algorithms, we have observed classification accuracy of up to 94.67% using the mHealth dataset and 95.12% on the ScientISST MOVE dataset. Other performance indicators, such as recall, precision, and F1 score, also performed well.

**Discussion:**

Overall, integrating a machine learning model with bio-signals provides an enhanced ability to interpret complex real-time patient health monitoring for personalized care and overall smart healthcare.

## 1 Introduction

In today’s modern world, smart healthcare management using advanced wearable gadgets plays an essential role in monitoring and predicting human health, especially in diagnosed patients for specialized healthcare (Riedel et al., 2008; Ogbuabor and La, 2018; Singh et al., 2023). Integrating bio-signals with embedded AI support for continuous monitoring and predicted specialized healthcare is important in today’s modern world (Han et al., 2023; Ahmed et al., 2023). Despite significant improvements in efficient health monitoring using wearable sensors, researchers are still working to improve and analyze complex physiological and vital signals (Bobade and Vani, 2020) for better and real-time health monitoring and predicting health degradation. Existing machine learning-based methodologies suffer from data variability and heterogeneity in data monitored using bio-medical signals (Yin et al., 2024; Guo et al., 2025) from daily life human activities. To address this issue, we have integrated biomedical signals from two extensive datasets, i.e., the mHealth dataset and the ScientISST MOVE dataset. In the mHealth dataset, which tracks vital signs with body movement, from the ScientISST MOVE dataset, a diverse range of bio-signals (Abdulmalek et al., 2022) are collected during their daily life movements and activities. This integration helps us with a wide-range analysis using complex physiological signals during daily life activities so that personal healthcare management is ensured using machine learning-based methodologies. In this study, our main focus is on novel applications and extensive validation of the existing methodologies for analyzing bio-signal for efficient health monitoring solutions, rather than introducing a new machine learning technique. The main contribution of this study is to address the gap between theoretical bio-signal analysis and a practical approach. In this study, we have selected three sensors: ECG (Electrocardiogram), EMG (Electromyography), and Accelerometer. The reason for selecting these three sensors is due to their commonalities with the selected two datasets, so that the consistency in data collection is ensured. For comparison and validation, it is also very important that the sensors are the same with the same data patterns for high accuracy, relevance, and robustness. Initially, after collecting raw signals from sensors, the noise from these signals was denoised. In step two, fixed windowing and segmentation techniques were used for data organization and efficient extraction of key features from signal data. In step three, which is most important, we have proposed a special feature block for ECG, EMG, and Accelerometer sensors. For an appropriate feature vector to train the model, the Linear Discriminant Analysis (LDA) technique is utilized. Finally, our proposed Deep Belief Network is cross-validated to evaluate the performance of the system. In the case of the mHealth dataset, our proposed system accuracy is 94%, while on the ScientISST MOVE dataset, accuracy is 95%, which is more than the mHealth dataset. The following are the contributions of our study.

**FIGURE 1:**
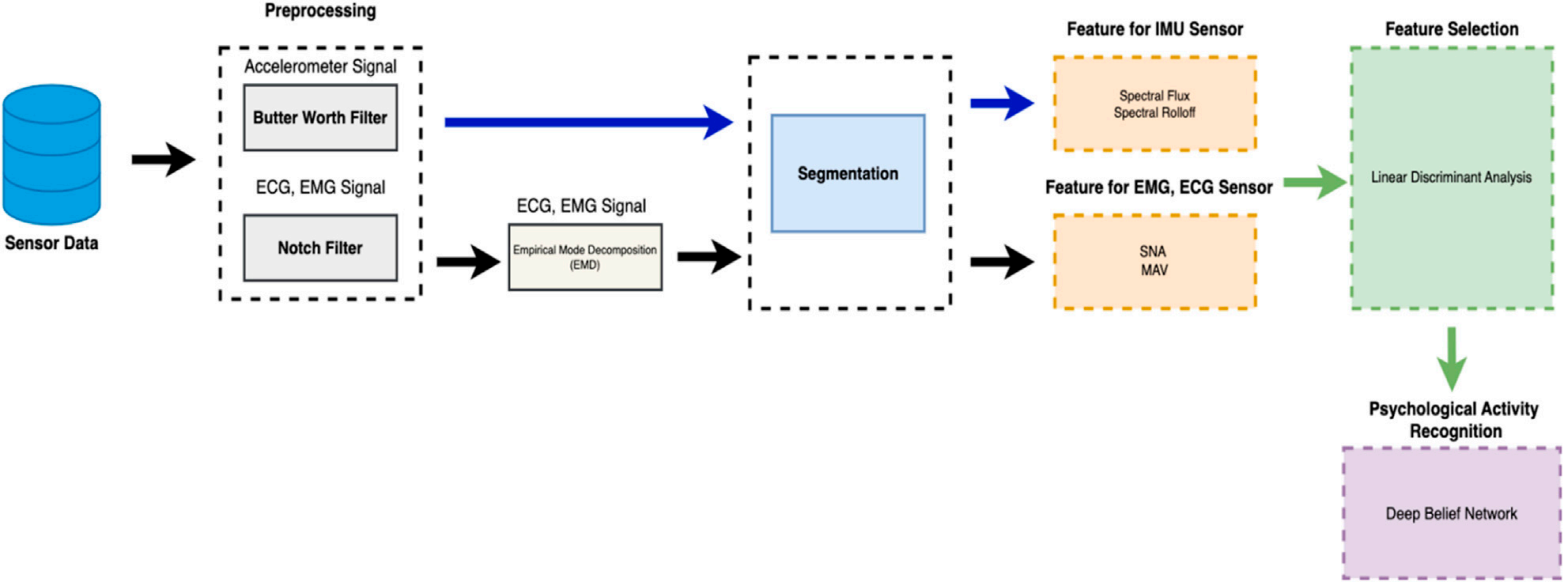
The architecture of the proposed system.

• Enhanced Integration of Signal Filtration Methodologies: We developed tailored signal filtration techniques to correct errors from sensor settings and orientation changes in wearable devices. Using this approach, data reliability for ECG, EMG, and IMU sensors has significantly improved, which is most suitable for its usage in real-world scenarios.
• Optimized Parallel Processing Framework: In this study, we have used an optimized parallel framework for simultaneous feature extraction from multimodal bio-signal, to manage real-time synchronous processing across various bio-signal types. This process significantly enhances activity recognition and signal monitoring of vital and other physiological signals.
• Hybrid Multidomain Feature Extraction: In our proposed system, we have adopted a hybrid approach by integrating time domain, frequency domain, and wavelet domain analysis of bio-signal for better accuracy, which ultimately improved health monitoring compared to single domain-based analysis.
• Application of EMD and SNA in Novel Contexts: In this study, we formulated Empirical Mode Decomposition (EMD) and Synergy Analysis (SNA) to analyze EMG and ECG signals monitored during daily activities. Due to its unique application, we explore complex aspects of bio-signals for enhanced insight analysis. The architecture of Proposed system can be seen in Figure 1.

## 2 Literature review

The study presented by (Alqarni, 2021), discusses an errorless data fusion (EDF) technique designed to enhance the accuracy of posture recognition using handheld wearable sensors (WSs) deployed in smart healthcare systems. This technique is used for monitoring the movement patterns of patients at various time intervals, followed by feature analysis using a random forest classification algorithm. The classification process efficiently identifies classification errors across different time intervals. This method emphasizes recurrent analysis, where conditional training based on previous errors is utilized to improve recognition accuracy. Key steps include data acquisition from wearable sensors, feature extraction based on integrity, chaining, and data patterns, and error reduction through classification. The performance metrics include accuracy, fusion error, and detection time against existing methods. The limitations include relying on data from wearable sensors, which may not capture the complete spectrum of patient movements and behaviors, potentially limiting the scope of posture detection. Similar to (Paraschiakos et al., 2020), the researchers conducted the GOTOV study, involving 35 participants performing 16 activities while wearing sensors on various body locations. They developed an activity ontology and utilized a combination of the Accordion algorithm, Random Forest, and LARA (Learning Activity Recognition Models Accurately) have been used to ensure high accuracy in activity classification. The study emphasized the effectiveness of specific sensor setups, particularly the combination of ankle and wrist accelerometers, for accurate activity recognition. They also validated the AR models in free-living conditions and made the GOTOV dataset publicly available. Some activities in the study were performed for varying time slots, leading to an unbalanced dataset. Moreover, in the case of an ideal scenario, while detecting more signal variation through repetitive and randomized activity performance was not fully gathered, hypothetically affecting the robustness and applicability of the AR models to diverse real-world scenarios. Another similar study conducted by (Demrozi et al., 2020) employs a machine learning-based approach for classifying accelerometer data to predict and recognize Freezing of Gait (FoG) in Parkinson’s Disease (PD) patients. The study employs tri-axial accelerometer sensors (Zheng et al., 2022; Zhang et al., 2023) attached to the back, hip, and ankle of patients. These sensors collected data at a sampling frequency of 65 Hz. The researchers used the k-nearest neighbor (k-NN) algorithm to classify the gait into three categories: pre-FoG, no-FoG, and FoG. The dataset used for the study was the DAPHNET benchmark suite, and the classification approach was validated through k-fold cross-validation. The study’s testing and application were primarily conducted in controlled environments. In (Hayano et al., 2020; Zyout et al., 2023), the authors explored the feasibility of detecting sleep apnea using photoplethysmography (PPG) signals from a wearable watch device. They engaged 41 patients undergoing diagnostic polysomnography (PSG) for sleep-disordered breathing and simultaneously recorded PPG data using a wearable watch. The study used an algorithm called auto-correlated wave detection with adaptive threshold (ACAT), originally developed for electrocardiogram (ECG) data, to analyze pulse intervals. This was done to detect cyclic variation of heart rate (CVHR) indicative of sleep apnea episodes. Key metrics like the apnea-hypopnea index (AHI) were used for comparison. The study’s approach involved comparing the frequency of CVHR detected by PPG with that detected by ECG (Hussain et al., 2023; Khan et al., 2023), assessing various statistical indices, and examining the algorithm’s sensitivity and specificity in sleep apnea screening. There are a few limitations in their study. Firstly, it focused solely on a single type of wearable watch, limiting the applicability of the findings across different devices. Secondly, the ACAT algorithm, initially designed for ECG data, might not be fully augmented for PPG data, theoretically affecting its accuracy. Additionally, subjects with conditions like continuous atrial fibrillation were excluded, which might limit the generalizability of the findings to all patients with sleep apnea. Finally, the study used time in bed as a denominator for calculating AHI, which could result in underestimating the severity of sleep apnea compared to calculations using total sleep time (Delmastro et al., 2020), The authors summarized a comprehensive study on cognitive and motor rehabilitation in frail older adults with Mild Cognitive Impairment (MCI), utilizing wearable physiological sensors and machine learning techniques. The methodology involved a randomized cross-over non-experimental study conducted in collaboration with a long-term care (LTC) facility. Participants, including frail older adults, were monitored during cognitive and motor rehabilitation sessions using two types of wearable sensors for heart rate, heart rate variability, and electrodermal activity (Liu et al., 2021; Yan et al., 2023; Zhang et al., 2023). Data were collected, pre-processed, and analyzed using various machine learning algorithms to evaluate stress response during the therapy sessions. The study also proposed a mobile system architecture for online stress monitoring, incorporating a Decision Support System (DSS) to personalize therapy (Zhao et al., 2025; Xing et al., 2024) based on detected stress levels. However, there are a few limitations. The research was conducted with a relatively small group of participants from a single LTC facility, which may limit the generalizability of the findings. The sample size and lack of diversity in the study population could impact the robustness and applicability of the results to a broader demographic. Another similar type of work conducted by Christian et al (Meisel et al., 2020), utilized deep learning techniques on data collected from wristband sensors worn by epilepsy patients. These wristbands continuously monitored several physiological parameters, including electrodermal activity, body temperature, blood volume pulse, and actigraphy (Díaz and Kaschel, 2023). The data were obtained from 69 patients during long-term, in-hospital monitoring, amounting to over 2,311 h and encompassing 452 seizures. The team employed a leave-one-subject-out cross-validation approach for their analysis, using long short-term memory (LSTM) networks, known for their efficacy in handling time series data. Data preprocessing included down-sampling to a uniform rate and filtering, followed by training the LSTM networks on matched pre- and interracial data segments. However, the relatively short-term duration of recordings, limited to just a few days per patient, might not have captured the full range of seizure characteristics and patterns.

### 2.1 Signal filtration and noise removal

Removing noise (Malik et al., 2023) is an important aspect of accurate monitoring and predictions. So, signal filtering is required before its processing. In this study, we have used advanced signal filtration methods to enhance the accuracy of multimodal bio-signals. The main purpose is to mitigate noise and interference so that the integrity and reliability of data are ensured for subsequent analysis. The bandpass filter (Agnuski et al., 2022) is employed to maintain signal frequencies within a defined range and attenuate frequencies that are beyond the defined range. This phase is specifically required and important for EMG and ECG signals so that meaningful signal information within the frequency range is ensured. This bandpass filter is formulated to adjust a range of frequencies between a lower frequency $f_{L}$ and a higher frequency $f_{H}$ to pass through while attenuating frequencies outside this range. Following is its mathematical representation. Equation 1 represents the mathematical form for the band-pass filter.

$$H\left(f\right)=\left\{\begin{array}{c} 1,if\;f_{L}\leq f\leq f_{H}\\ 0,otherwise \end{array}\right.$$ (1)

In this case, $H\left(f\right)$ It is considered the frequency response of the filter, *f* is the frequency, $f_{L}$ and the lower range frequency and $f_{H}$ is the higher range frequency. This model successfully isolates the signal within the desired frequency band and the attenuating components.

Moreover, regarding the issue of power line interference, which is commonly observed at 50 Hz (or 60 Hz in certain regions), we have implemented a notch filter to address this issue (Cengiz et al., 2022), This specific filter is formulated to attenuate a narrow band of frequencies around the specified notch frequency (50 Hz). The mathematical formulation is as follows:

$$H\left(f\right)=\left\{\begin{array}{l} 0,if\;f=f_{N}\\ 1,otherwise \end{array}\right.$$ (2)

In Equation 2 $H\left(f\right)$ is the frequency response, f is the frequency, and $f_{N}$ is the notch frequency.

In the case of the ECG Signal, we have applied a bandpass filter to isolate the frequency components specifically in the heart’s electrical activity within a specific range suitable for ECG analysis. Here, the notch filter is used to remove power line interference, typically at 50 Hz or 60 Hz, which is considered a source of noise in ECG measurements. For the EMG Signal, the bandpass filter was used to capture the frequency range where muscle electrical activity is most prominent. EMG signals have their most significant information content in a specific frequency band, and the bandpass filter helps in isolating these frequencies. The notch filter was also applied to the EMG signals to remove electrical noise, especially the interference from power lines, which can significantly affect the quality of EMG recordings.

For Accelerometer, we have used a low-pass Butterworth filter, which is widely used in similar studies (Ding et al., 2023; Wang et al., 2023; Hu et al., 2023). The Butterworth low-pass filter is mostly used for noise reduction in time-series data. Its mathematical representation is as follows. The mathematical representation for butterworth low-pass filter is as follow:

$$X\left(y\right)=\frac{1}{1+{\left(\frac{y}{j\cdot w_{c}}\right)}^{2_{N}}}$$ (3)

**FIGURE 2:**
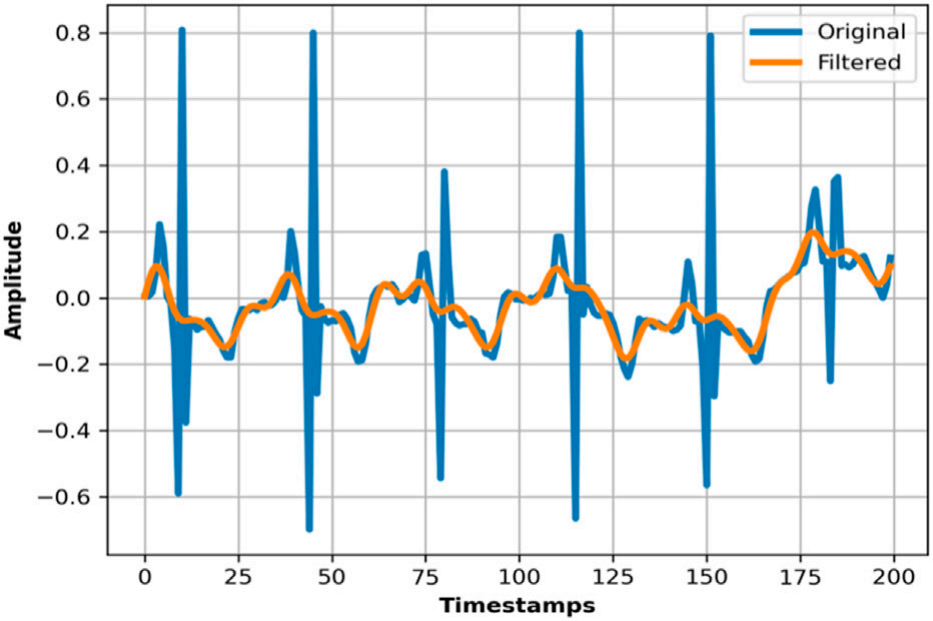
Actual vs. Filtered ECG signal using the mHealth dataset.

In this case, the left-hand side, i.e., $X\left(y\right)$ represents the transfer function of the Butterworth filter, *y* is the complex frequency variable, *j* is the imaginary unit, and $w_{c}$ is the angular frequency, and N is the order of the filter, which specifies the sharpness of the frequency response curve. In our proposed model, the parameters are carefully selected to strike a balance between noise reduction and signal preservation. The value of *N = 4* was selected to achieve effective noise reduction while minimizing signal distortion. The cutoff frequency was set to $w_{c}$ = 0.3 Hz, ensuring that high-frequency noise components are attenuated while preserving the essential low-frequency dynamics of human activities. The pictorial presentation of the bandpass filter is visible in Figure 2.

### 2.2 EMD (empirical mode decomposition for EMG and ECG sensor)

**FIGURE 3:**
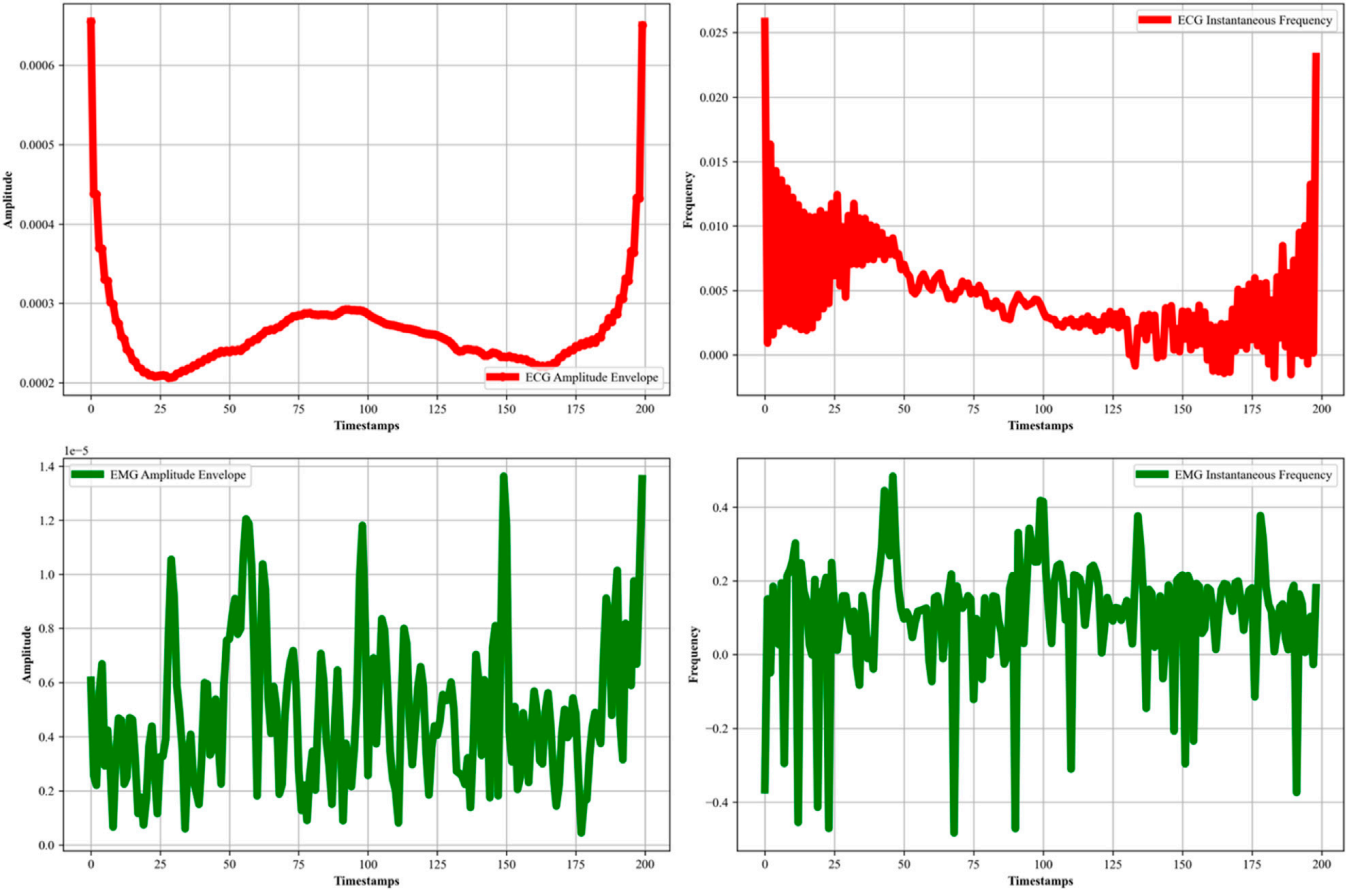
EMD plotted for EMG and ECG signal over the ScientISST MOVE dataset.

Decomposition of the EMG and ECG signal is further required to extract the meaningful insights and features of the like amplitude envelopes (intensity) and instantaneous frequency temporal dynamics. This decomposition helps us to extract cardiac and muscular meaningful insights for the most effective healthcare management. So, in this study, we also applied Empirical Mode Decomposition (EMD) (Hu et al., 2020; Centeno-Bautista et al., 2023) to both the ECG and EMG signals. The EMD method is used for analyzing non-linear and non-stationary data, which is beneficial for accurate physiological signal characterization. This process involves decomposing each signal into a set of Intrinsic Mode Functions (IMFs) through an iterative ‘sifting’ procedure. Each of the IMF represents a simple oscillatory mode extracted from the original signal, and together, they reconstruct the signal’s full information content. By applying the Hilbert transform (Goecks et al., 2020; Mishra and Bhusnur, 2022) to these IMFs, we further obtained the amplitude envelope and instantaneous frequency for each signal, as shown in Figure 3. In the case of ECG signals (Li et al., 2024; Tao et al., 2023), the amplitude envelope graph reveals the signal’s overarching oscillatory magnitude, while the instantaneous frequency graph represents a detailed analysis of the heart rate variability and cardiac dynamics. The EMG signal’s amplitude represents the muscular activity, while the instantaneous frequency represents temporal evolution and fatigue. In Equation 4, the EMD process can be described mathematically as:

$$x\left(t\right)=\sum_{n=1}^{N}IMF_{n}\left(t\right)+r_{N}\left(t\right)$$ (4)

Here, the variable x (t) is the actual signal, while $IMF_{n}\left(t\right)$ represents intrinsic mode functions, and $r_{N}\left(t\right)$ is the residue. The result obtained after applying EMD can be seen in Figure 3.

### 2.3 Feature extraction for EMG and ECG sensor

In the feature extraction phase of our analysis, we focused on deriving insightful metrics from the EMG and ECG signals that could robustly characterize physiological activities. For EMG signals, Mean Absolute Value (MAV), Power Spectral Density (PSD), and Waveform Length (WL) are the key features.

#### 2.3.1 Muscle synergy analysis

The purpose of Muscle Synergy Analysis (Chaddad et al., 2023; Wang and Shang, 2023; Kumar et al., 2023), is to understand how muscle coordination is performed for various tasks. As per medical science, it is assumed that the central nervous system overall controls multiple muscles by activating them in groups, or synergies, rather than individual control. This analysis is performed using a computational technique known as Non-negative Matrix Factorization (NMF).

This model NMF decomposes the preprocessed EMG data matrix *V* into two lower-rank non-negative matrices *W* and *H*, where *V ≈ WH*. where *V* is of size *m × n* (with *m* representing the number of muscles and *n* the number of time samples), *W* is of size *m × k* (with *k* representing the number of synergies), and *H* is of size *k × n*. The matrix *W* contains the synergy vectors, indicating the weight of each muscle’s contribution to a synergy, while *H* contains the temporal activation patterns of each synergy over time. This decomposition is used to identify common patterns of muscle activation (Juan and Greiner, 2021) (synergies) across different activities.

**FIGURE 4:**
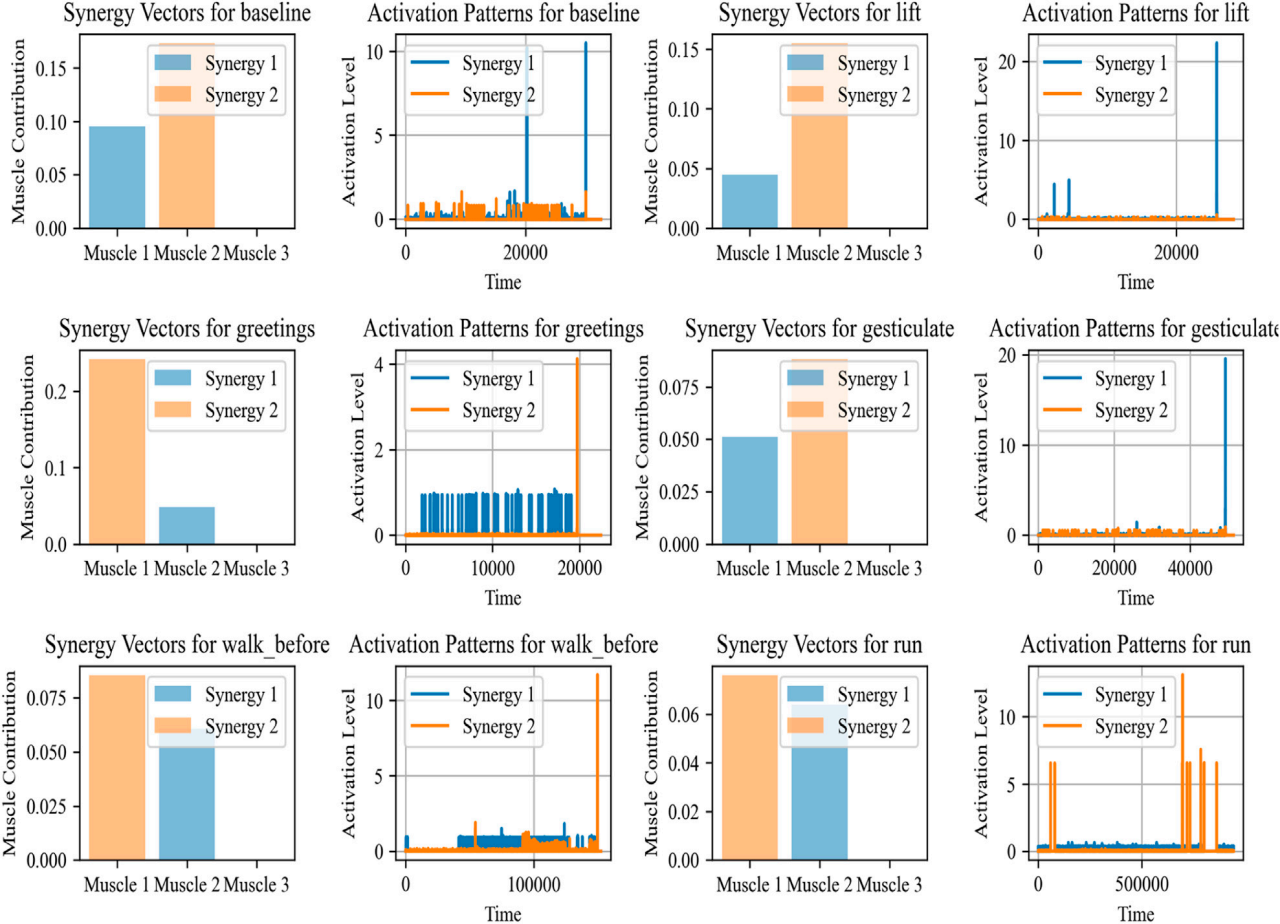
The synergy, along with activation patterns for EMG sensors for different activities calculated over the ScientISST MOVE dataset.

Muscle Synergy Analysis has various applications (Jabeen et al., 2023). For example, in the rehabilitation of patients with motor impairments, such an analysis of muscle synergies can guide the development of specialized therapies. Also in sports, it is beneficial to optimize training regimens by understanding muscle coordination and fatigue. Additionally, it can also be beneficial in the design of assistive devices such as exoskeletons or in controlling robotic prosthetics by mimicking the natural synergistic patterns of muscle activation (Rodrigues et al., 2022; Zhou and Zhang, 2022). Figure 4 demonstrates the synergy vectors and activation patterns for baseline, lift, greetings, gesticulate, walk before, and run. The synergy vectors and their corresponding activation patterns show how muscle groups are coordinated in our daily life activities.

#### 2.3.2 EMG mean absolute value (MAV)

The following is the mathematical model of MAV, which formulates muscle contraction strength (Islam et al., 2023) by taking the average of the absolute amplitude of the EMG signal.

$$MAV=\frac{1}{N}\sum_{i=1}^{N}1\left|x_{i}\right|$$ (5)

**FIGURE 5:**
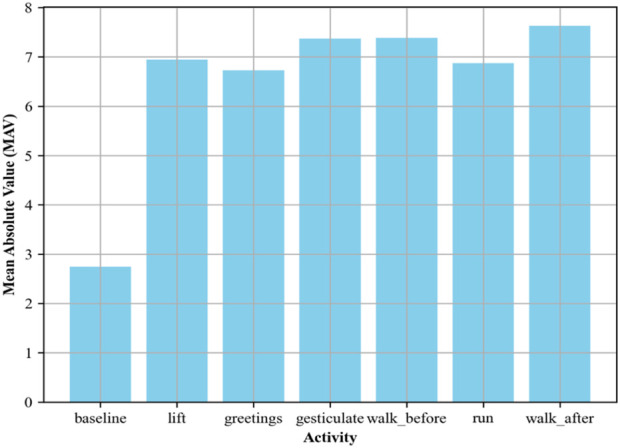
MAV plotted for the EMG signal over the ScientISST MOVE dataset.

In Equation 5, the mathematical model of MAV, where $x_{i}$ is the EMG signal amplitude at the *i*th sample, while N is the total number of samples. This formulation is useful in recognizing muscle activation patterns, as it correlates with the force generated by the muscle. Figure 5 represents the calculations.

#### 2.3.3 EMG power spectral density (PSD)

Similarly, PSD is used to analyze the distribution of power across various frequencies in EMG so that insights regarding fatigue may be identified. Following as the formulation of PSD in Equation 6 (Alam et al., 2020; Zhang et al., 2022; Zhu et al., 2023) by using the method of the Fast Fourier Transform (FFT):

$$PSD\left(f\right)={\left|FFT\left(x\left(t\right)\right)\right|}^{2}$$ (6)

**FIGURE 6:**
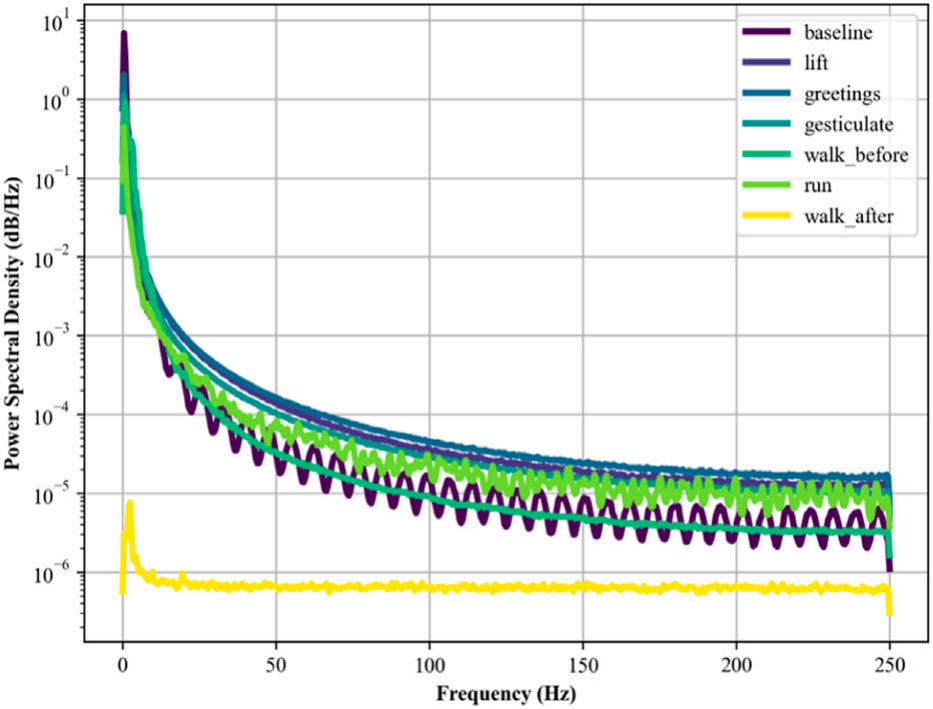
PSD plotted for EMG signal over the ScientISST MOVE dataset.

Here, *x(t)* is the EMG signal as a function of time, and *f* represents frequency. PSD is crucial in human physiology recognition and reflecting different muscle activities. Its calculations are in Figure 6.

#### 2.3.4 EMG waveform length (WL)

Waveform Length is used to quantify the complexity of the EMG signal (Sei-ichi et al., 2023; Hu et al., 2024) with respect to time, indicating temporal variability (Arquilla et al., 2022). Following is the mathematics. Waveform length can be mathematically represented in Equation 7 as:

$$WL=\sum_{i=1}^{N-1}\left|x_{i+1}-x_{i}\right|$$ (7)

**FIGURE 7:**
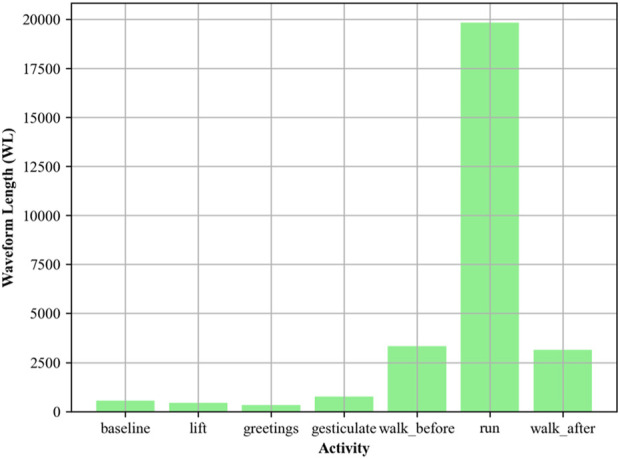
Waveform Length plotted for different activities using EMG signal over the ScientISST MOVE dataset.

Where $x_{i}$ and $x_{i+1}$ are the consecutive EMG signal amplitudes. WL can be particularly helpful in identifying changes in muscle contraction patterns and is, therefore, valuable in activity recognition tasks. Figure 7 illustrates the EMG signal vs activity.

### 2.4 Feature extraction for IMU sensor

#### 2.4.1 Spectral rolloff

In this phase, we are calculating the spectral rolloff (Pires et al., 2019) of the accelerometer data to quantify the distribution of higher frequency components (Ku et al., 2018) present within the received signal. Here, the application of the Fast Fourier Transform (FFT) on each segmented window of time-domain accelerometer data (Junaid et al., 2022; Wen et al., 2023) is a crucial step in shifting the perspective from time to frequency, enabling the analysis of the frequency content of the signals. Following the FFT, the Power Spectral Density (PSD) of each window is computed. The PSD is a measure that indicates the power present in each frequency component of the signal, providing a comprehensive picture of how the energy of the signal is distributed across different frequencies.

**FIGURE 8:**
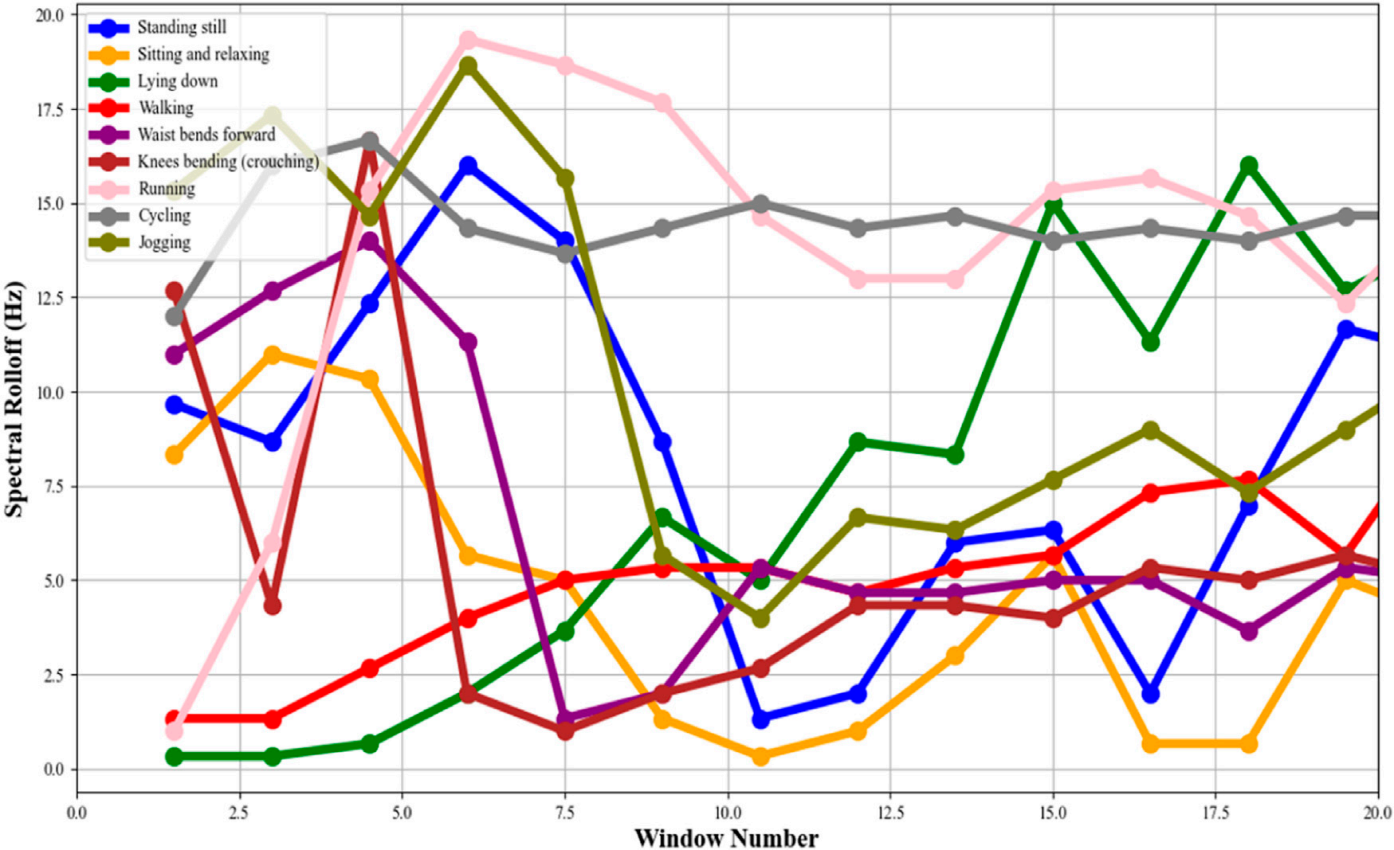
Spectral roll off points plotted for the accelerometer signal over the mHealth dataset.

The core of this process lies in calculating the spectral rolloff. It is a measure that captures the frequency below which a defined percentage of the total spectral energy is contained. Typically, this threshold is set around 85%. Mathematically, if we denote *F* as the set of frequencies in the power spectrum and *P(f)* as the power at frequency *f*, the spectral roll-off *R* is defined as the lowest frequency for which the cumulative sum of the power from the lowest frequency up to *R* equals or exceeds a certain percentage (e.g., 85%) of the total spectral power. This can be represented in Equation 8 as:

$$\sum_{f=0}^{R}p\left(f\right)\geq k\cdot \sum_{f\epsilon F}p\left(f\right)$$ (8)

where k is the rolloff percentage. For example, in health and fitness trackers, these features enhance the accurate categorization of user activities, enhancing the device’s ability to monitor and provide feedback on physical activity levels. Similarly, in real settings, the analysis of these features can offer critical insights into a patient’s mobility and activity patterns, which is invaluable in rehabilitation programs and disease progression monitoring. The spectral rolloff calculated for different activities can be seen in Figure 8.

#### 2.4.2 Spectral flux

This model is used to calculate and measure the changes in the power spectrum of a signal (Ma et al., 2022) from one frame to the next, providing a further understanding of how the signal’s frequency content varies. To analyze the spectral flux, the Fast Fourier Transform (FFT) is applied to segments of the accelerometer data to convert it from the time domain to the frequency domain, resulting in a series of power spectra. The spectral flux (Alsaify et al., 2022; Du et al., 2019; Gao et al., 2019) is then calculated as the sum of the squared differences between the magnitudes of the power spectra of consecutive segments. Numerically in Equation 9, if *P_n_(f)* represents the power spectrum at frequency *f* of the *n*th segment, the spectral flux *SF_n_* between segment *n* and segment *n−1* is defined as:

$${SF}_{n=}\sum {\left(P_{n}\left(f\right)-P_{n-1}\left(f\right)\right)}^{2}$$ (9)

**FIGURE 9:**
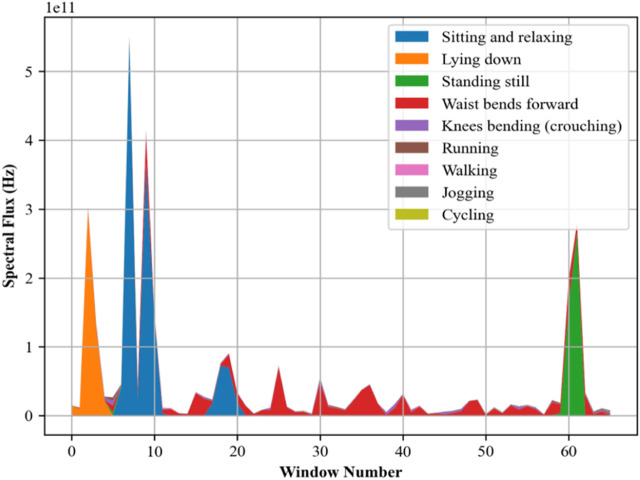
Spectral flux plotted for the accelerometer signal over the mHealth dataset.

Spectral flux (Liu A. et al., 2022; Yao et al., 2023) assists as a critical feature in the recognition of different physical activities and the assessment of posture characteristics in movement investigations. Capturing dynamic changes in movement helps in the early detection of deviations from normal patterns that could indicate health issues and helps in preventative healthcare monitoring and prediction. Figure 9 presents the Spectral flux calculated for different activities.

### 2.5 Linear discriminant analysis (LDA)

Linear Discriminant Analysis (LDA) is applied to reduce the dimensions of a feature vector (Arshad et al., 2022; Qu et al., 2023; Zhang et al., 2022), The goal of LDA is to project the data onto a lower-dimensional space with good class-separability to avoid overfitting (“curse of dimensionality”) and also reduce computational costs (Strzelecki and Badura, 2022; Zhao et al., 2022). Mathematically in Equation 10, LDA seeks to find a projection that maximizes the between-class variance $\sigma_{between}^{2}$ while minimizing the within-class variance $\sigma_{within}^{2}$ , hence maximizing the ratio:

$$Q\left(w\right)=\frac{w^{t}s_{b}w}{w^{t}s_{w}w}$$ (10)

**FIGURE 10:**
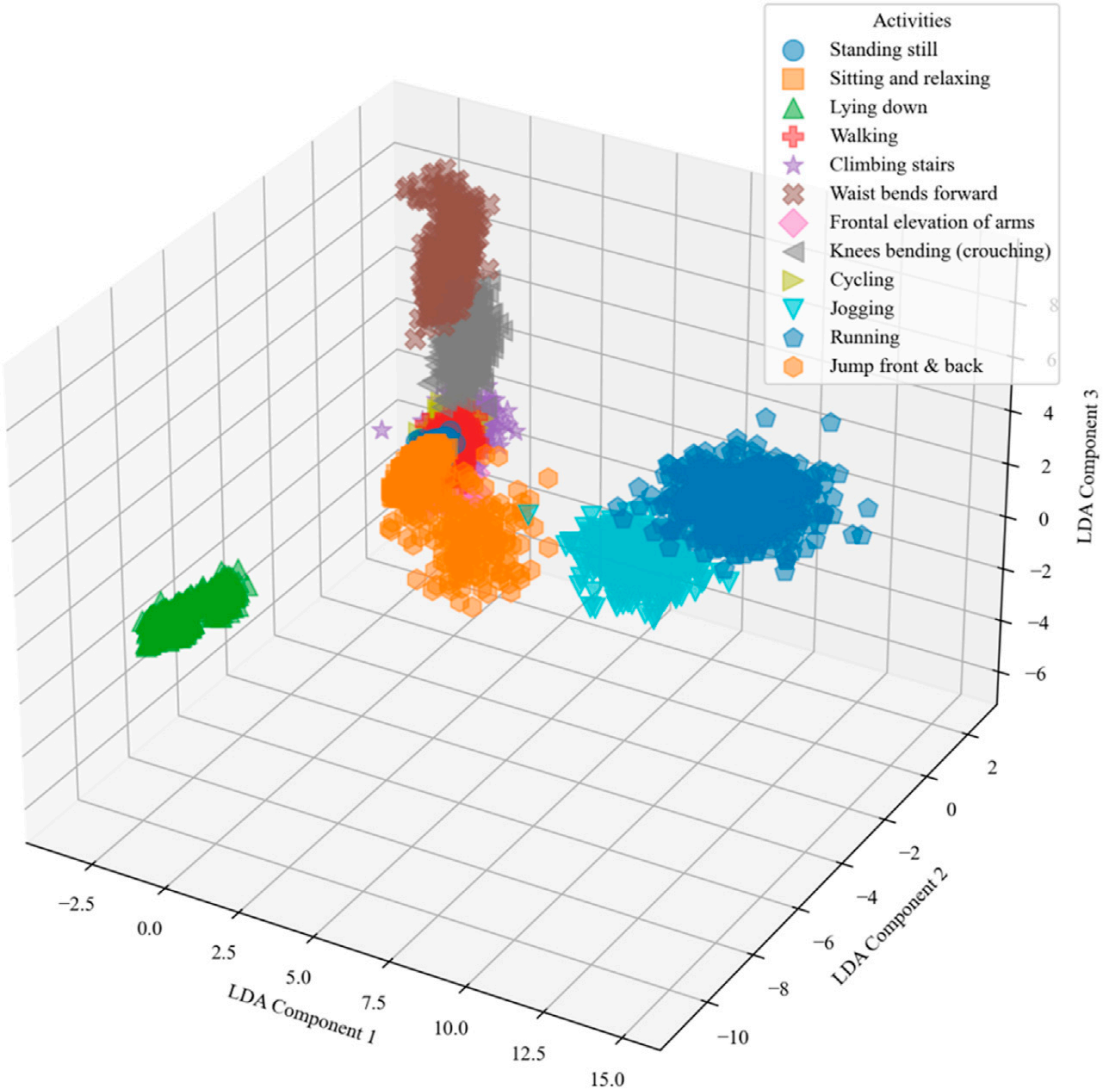
The LDA was performed over the mHealth dataset.

Where *w* is the vector that defines the projection direction, $s_{b}$ is the between-class scatter matrix, and $s_{w}$ is the within-class scatter matrix (Qu et al., 2023; Su et al., 2023). The optimal *w* is found as the eigenvector of $s_{w}^{-1}s_{b}$ corresponding to the largest eigenvalue. Figure 10 represents the analysis of LDA on the mHealth dataset.

### 2.6 Activity recognition

**FIGURE 11:**
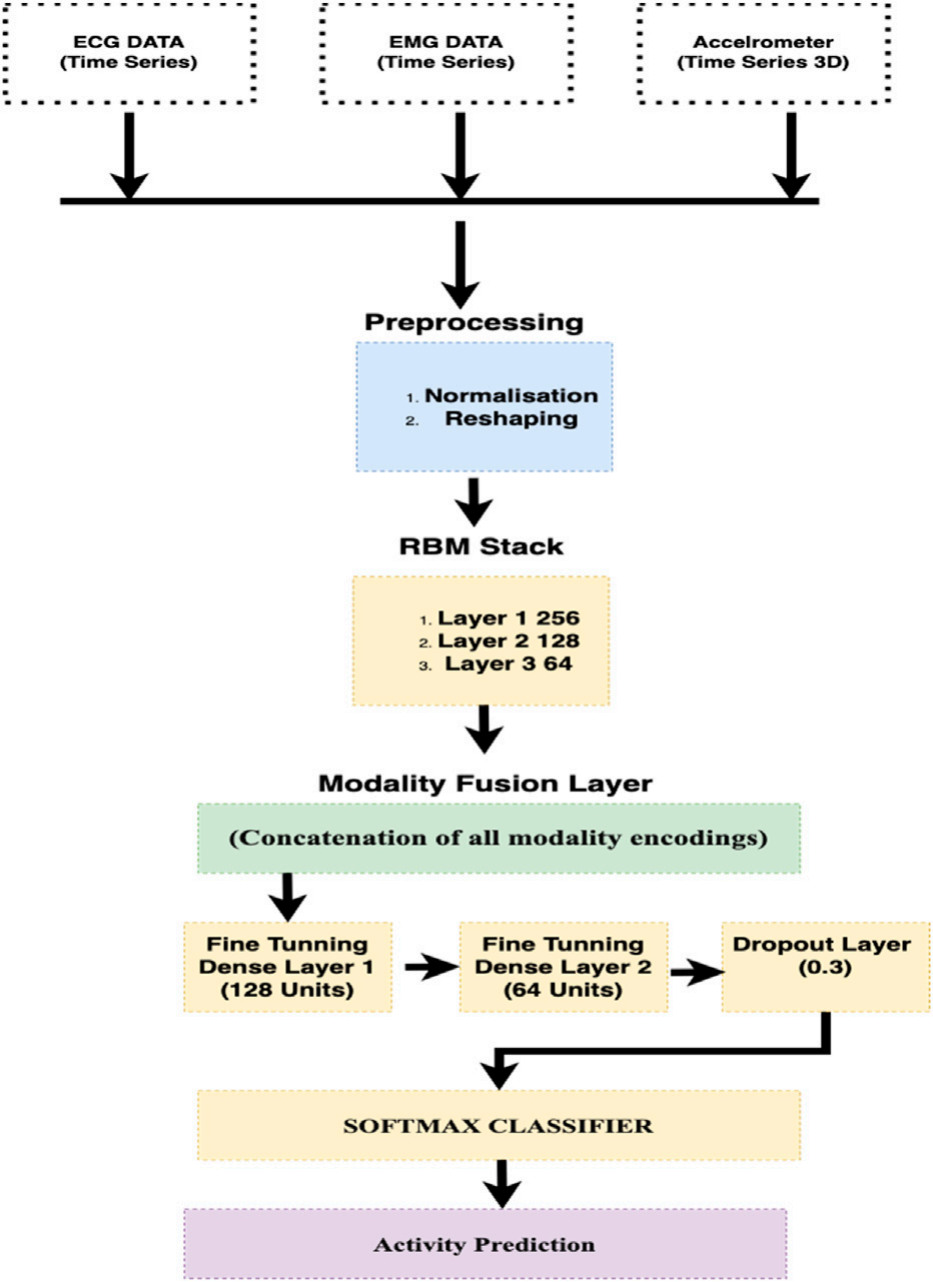
Proposed BDN architecture.

In this study, we used an advanced machine learning model, i.e., Deep Belief Network (DBN) (Murad and Pyun, 2017; Liang et al., 2018), to analyze the multi-dimensional data resulting from wearable sensors. This model includes multiple layers of stochastic, latent variables, allowing it to efficiently handle the diverse and complex nature of sensor data, which often includes accelerometers, ECG, and EEG signal measurements. Each layer of the DBN is intended to extract higher-level features (Qi et al., 2022; Wang et al., 2023) from the raw sensor data, simplifying the recognition of complex patterns associated with numerous human activities. The early layers detect the basic motion attributes, such as direction and speed, while the bottom layers are responsible for integrating these attributes into recognizable activities. This model is effective in distinguishing between activities with similar motion profiles but different contextual variations. Our proposed DBN model provides a robust and accurate classification of human activities. Figure 11 shows the diagram of DBN.

### 2.7 Cross-validation methodology

In this study, a subject-wise k-fold cross-validation method is implemented to evaluate the generalizability of our predictive techniques. The purpose of our cross-validation model is to ensure that each subject’s data is solely allocated to either the training or the testing phase so that the data leakage is eliminated. During this process, the subjects were randomly partitioned into k groups to preserve a representative distribution of the dataset’s variability (Rastogi and Mehra, 2013; Wang et al., 2022). The model was then trained on k-1 groups, tested on the remaining groups, and repeated on all groups. We further calculated the performance metrics such as accuracy, precision, recall, and the F1 for each fold and then accumulated them to provide an overarching evaluation of the model’s predictive performance. The following are the steps.

• Partitioning: The entire set of subjects *S* is divided into *k* distinct groups $S_{1,}S_{2\ldots \ldots \ldots }S_{k}$ such that each group $S_{i}$ contains approximately $\frac{1}{k}$ total subjects, ensuring no overlap in subjects between any two groups.
• Validation Cycle: For each fold *i*, the model is trained on the union of all groups except $S_{i}$ those denoted as SSi , and test on $S_{i}$ . This is mathematically represented in Equation 11 as:

$$\text{Train on }\underset{j\neq 1}{⋃}S_{j},\text{Test on }S_{i}$$ (11)

• Performance Metrics: After training and testing, performance metrics such as accuracy (ACC), precision (P), recall (R), and F1 score are calculated. These metrics for each fold are defined in Equation 12 as:

$${Acc}_{i}=\frac{\text{True}\;\text{Positives}+\text{Ture}\;\text{Negatives}}{\text{Total}\;\text{Samples}},P_{i}=\frac{\text{True}\;\text{Positives}\;}{\text{True}\;\text{Positives}+\text{False}\;\text{Positives}},R_{i}=\frac{\text{True}\;\text{Positives}\;}{\text{True}\;\text{Positives}+\text{False}\;\text{Negatives}},{F1}_{i}=2\times \frac{P_{i}\times R_{i}}{P_{i}+R_{i}}$$ (12)

• Aggregation: The overall performance across all fold is determined by averaging these matrics using the Equations 13, 14 and 15.

$$\text{Mean Acc}=\frac{1}{k}\sum_{i=1}^{k}{\text{Acc}}_{i}$$ (13)

$$\text{Mean Precision}=\frac{1}{k}\sum_{i=1}^{k}P_{i}$$ (14)

$$\text{Mean Recall}=\frac{1}{k}\sum_{i=1}^{k}R_{i}$$ (15)

## 3 Performance evaluation

The standard performance metrics are used for measuring the efficiency and evaluation of our proposed model. For example, confusion matrices, precision, recall metrics, F1 scores, and Receiver Operating Characteristic (ROC) curves collectively demonstrated their effectiveness.

### 3.1 Dataset description

As discussed earlier, we have used two well-known datasets, i.e., the mHealth dataset (Banos et al., 2014), which is used for the development and evaluation of mobile health monitoring and tracking technologies. This dataset consists of multidimensional sensor data collected via wearable gadgets, including accelerometers, gyroscopes, and magnetometers, having a broad range of human daily life activities data. This heavy dataset covers different physical activities including, standing still, Sitting and relaxing lying down, and so on, and is available for research work.

Similarly, the ScientISST MOVE dataset is another comprehensive collection of multimodal bio-signals, collected in the context of everyday life activities, compiled by (Areias Saraiva et al., in 2023) using wearable bio-signal, acquired from 17 healthy volunteers as they engaged in a variety of common activities, such as walking, running, and gesturing. This dataset consists of electrocardiogram (ECG), electrodermal activity (EDA), and photoplethysmography (PPG) signals, alongside electromyography (EMG) from the biceps, wrist temperature (TEMP), and actigraphy data from the chest and wrist (ACC).

## 4 Results and analysis

This section illustrates the experimental analysis and evaluation of our proposed model using different experiments. For measuring the performance, we have used standard evaluation metrics such as the confusion matrix, precision, recall, F1 score, and receiver operating characteristic (ROC) curve. The following sub-sections present the details.

### 4.1 Experiment 1: confusion matrix

In this experiment, we design the confusion matrix for both datasets. This matrix withdraws numerical results are depicted in Tables 1, 2 for the mHealth and ScientISST MOVE Datasets.

**TABLE 1:** Confusion matrix calculated over the mHealth dataset.

Obj. Classes	SS	SR	LD	Walking	CS	WBF	FEOA	KB	Cycling	Jogging	Running	JFB
SS	**0.97**	0.01	0.00	0.00	0.00	0.01	0.00	0.00	0.00	0.00	0.00	0.00
SR	0.01	**0.95**	0.00	0.00	0.00	0.02	0.00	0.00	0.00	0.01	0.00	0.01
LD	0.00	0.00	**0.97**	0.02	0.00	0.00	0.00	0.00	0.00	0.00	0.00	0.01
Walking	0.00	0.00	0.00	**0.99**	0.00	0.00	0.00	0.00	0.00	0.00	0.01	0.00
CS	0.02	0.00	0.00	0.00	**0.92**	0.00	0.00	0.04	0.00	0.00	0.01	0.01
WBF	0.00	0.00	0.02	0.00	0.01	**0.91**	0.03	0.00	0.00	0.03	0.00	0.00
FEOA	0.00	0.00	0.00	0.00	0.00	0.00	**0.98**	0.00	0.02	0.00	0.00	0.00
KB	0.00	0.03	0.00	0.00	0.00	0.00	0.00	**0.97**	0.00	0.00	0.00	0.00
Cycling	0.00	0.00	0.00	0.00	0.00	0.00	0.00	0.00	**0.96**	0.04	0.00	0.00
Jogging	0.00	0.00	0.00	0.00	0.00	0.02	0.00	0.00	0.00	**0.98**	0.00	0.00
Running	0.00	0.00	0.00	0.00	0.00	0.00	0.00	0.00	0.00	0.00	**1.00**	0.00
JFB	0.00	0.00	0.00	0.00	0.00	0.00	0.00	0.00	0.00	0.00	0.00	**1.00**
**Mean Accuracy = 94.67%**												

SS , standing still; SR , sit and relax; LD, lying down; CS, climbing stairs; WBF, waist bends forward; FEOA, frontal elevation of arms; KB, kneens bending; JFB, Jump front and back. The bold values represents the accuracy level for each activity, For example 0.97 means 97 percent of samples are correctly classified out of 100.

**TABLE 2:** Confusion matrix calculated over ScientISST MOVE dataset.

Obj. Classes	Bsln	Gst	Gtng	Lift	Run	W_a	W_b
Bsln	**0.94**	0.01	0.00	0.04	0.00	0.01	0.00
Gst	0.01	**0.89**	0.03	0.05	0.00	0.02	0.00
Gtng	0.03	0.00	**0.87**	0.02	0.04	0.03	0.01
Lift	0.02	0.00	0.00	**0.98**	0.00	0.00	0.00
Run	0.00	0.00	0.00	0.00	**1.00**	0.00	0.00
W_a	0.00	0.00	0.00	0.00	0.01	**0.99**	0.03
W_b	0.00	0.02	0.00	0.00	0.00	0.00	**0.98**
**Mean Accuracy = 95.12%**							

Bsln = Baseline, Gst = Gesticulate, Gtng = Greetings, W_a = Walk_after, W_b = Walk_before. The bold values represents the accuracy level for each activity, For example 0.97 means 97 percent of samples are correctly classified out of 100.

### 4.2 Experiment 2: precision, recall and F1 score

In this experiment, the proposed system is rigorously assessed. Detailed discussions are also provided on the specific implications of the system in certain areas. Table 3 represents the performance of the system using precison, recall and F1 score.

**TABLE 3:** Precision, Recall, and F1 score for mHealth and ScientISST MOVE Datasets.

Classes	mHealth			ScientISST MOVE		
Activities	Precision	Recall	F1 score	Precision	Recall	F1 score
Standing still	0.97	0.94	0.95	—	—	—
Sit and relax	0.83	0.92	0.90	—	—	—
Lying down	0.86	0.97	0.91	—	—	—
Walking	0.90	0.99	0.98	—	—	—
Climbing stairs	0.89	0.89	0.95	—	—	—
WBF	0.82	0.91	0.88	—	—	—
FEOA	0.92	0.98	0.95	—	—	—
Knees bending	0.83	0.96	0.89	—	—	—
Cycling	0.83	0.98	0.90	—	—	—
Joging	0.84	0.97	0.90	—	—	—
Running	1.00	1.00	0.98	—	—	—
JFB	1.00	1.00	1.00	—	—	—
Baseline	—	—	—	0.89	0.95	0.97
Gesticulate	—	—	—	0.81	0.94	0.90
Greetings	—	—	—	0.88	0.83	0.90
Walk_after	—	—	—	0.98	1.00	0.97
Lift	—	—	—	0.97	0.91	0.99
Run	—	—	—	1.00	1.00	1.00
Walk_before	—	—	—	0.98	0.95	1.00

WBF, waist bends forward; FEOA, frontal elevation of arms; JFB, Jump front and back.

#### 4.2.1 Discussion and analysis

Based on the results from experiment 1, the analysis of the health-related Human Activity Recognition (HAR) dataset, incorporating the mHealth and ScientISST MOVE datasets, shows an upright performance across various activities. The precision metric, which calculates the accuracy of the model in predicting an activity after recognition, varies significantly across different activities. For example, in the mHealth dataset, activities such as ‘Running’ and ‘JFB’ demonstrate accurate precision, indicating that the model’s predictions for these activities are highly reliable and robust. This enhanced precision is beneficial in personalized health monitoring, where accuracy matters a lot. In contrast, activities like ‘Sit and relax’ and ‘Climbing Stairs’ in mHealth show comparatively inferior precision. Similarly, in another dataset, i.e., ScientISST MOVE, activities such as ‘Lift’ and ‘Walk before’ show high precision, which is useful in scenarios like physical therapy where precise movements are desirable. Moreover, the recall, indicating the proposed model’s ability to correctly identify true instances of an activity, also shows inconsistency. For activities like ‘Walking’ in mHealth and ‘Walk_after’ in ScientISST MOVE, the recall is remarkably high, suggesting that the model is highly effective at detecting these activities when they occur. These findings are beneficial in fitness tracking and patient activity monitoring in specialized healthcare management systems, where missing out on such common activities could lead to imprecise health assessments. However, in a few activities like ‘WBF’ in mHealth reveal inferior recall, which could lead to under-detection in scenarios requiring continuous activity monitoring. The F1-score, a harmonic mean of precision and recall, provides a balanced view of the model’s overall performance. High F1 scores for activities like ‘Lying down’ in mHealth and ‘Baseline’ in ScientISST MOVE indicate the model’s effectiveness in accurately identifying and classifying these activities, which is vital in applications like sleep studies or relaxation therapy. However, the lower F1 scores in the case of activities indicate areas of improvement. For instance, activities with lower F1 scores might be less reliably tracked in contexts requiring detailed activity analysis, such as specialized fitness programs or advanced human-computer interaction systems as shown in Table 3.

### 4.3 Experiment 3: ROC (receiver operating characteristic curve)

**FIGURE 12:**
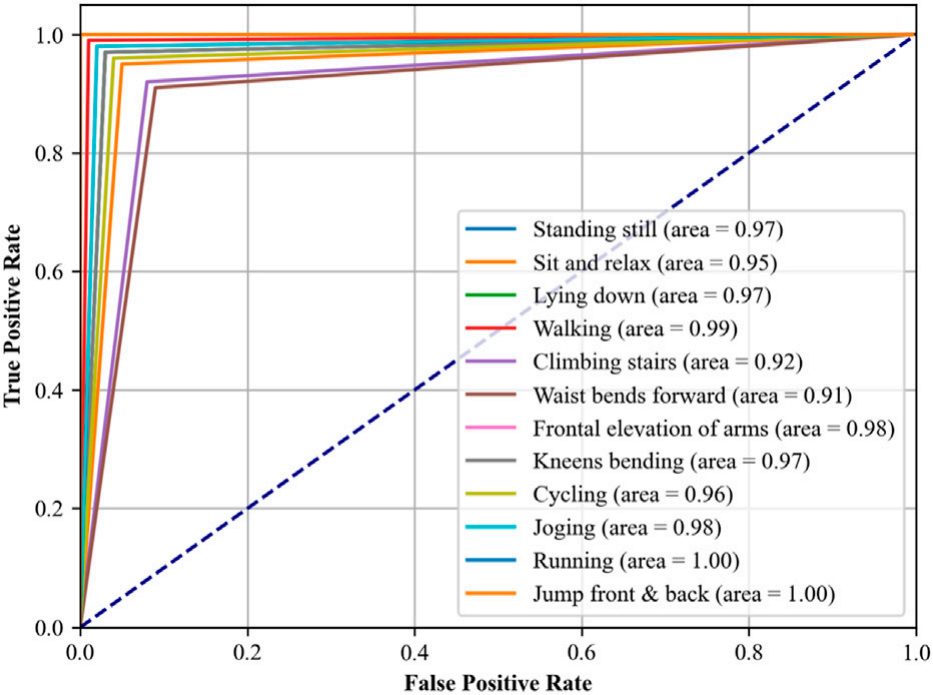
ROC curve plotted over mHealth Dataset.

**FIGURE 13:**
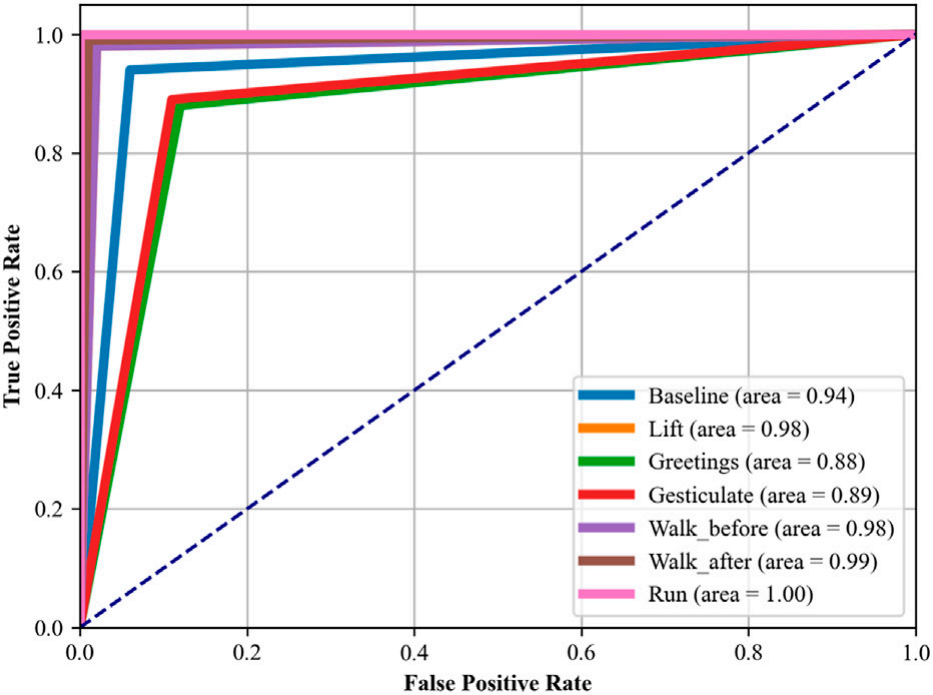
ROC curve plotted over the ScientISST MOVE Dataset.

In another experiment, i.e., Receiver Operating Characteristic (ROC) curves indicate how well our proposed DBN classifier can distinguish between altered health’s health-related activities. The area under the ROC curve (AUC) provides a single scalar value to indicate overall performance. The closer the AUC to 1, indicates better the performance in terms of distinguishing between the positive class (the specific activity) and the negative class (all other activities). The detailed analysis was conducted using a OneVsRest strategy with a DBN classifier. Each activity was treated as a separate binary classification problem, and the output was binarized to reflect the presence or absence of each activity class. The dataset was subdivided into training and test sets, where the classifier was trained in the first and used to predict class probabilities in the second part. Then we computed the false positive rate (FPR) and true positive rate (TPR) across the entire test set, which included data from all subjects across all folds, ensuring a complete evaluation of the classifier’s performance. These steps were carefully taken to ease any potential for data leakage and to provide a realistic portrayal of the classifier’s ability to generalize. The resulting ROC curves for each class are illustrated in Figures 12, 13, respectively, with the area under the curve (AUC) providing a scalar measure of performance.

#### 4.3.1 Discussion and analysis of ROC curve

In Figure 12, which assesses the mHealth dataset, activities such as ‘Standing still’, ‘Lying down’, and ‘Walking’ show near-perfect AUCs of 0.97, 0.97, and 0.99, respectively, indicating excellent model performance for these activities. ‘Running’ and ‘Jump front and back’ activities have perfect AUCs of 1.00, reflecting the model’s exceptional capability in recognizing these dynamic movements with high accuracy. Figure 13, analyzing the ScientISST MOVE dataset, demonstrates strong performance across different activities, with ‘Lift’ and ‘Walk_after’ activities having AUCs of 0.98 and 0.99, respectively, signifying high accuracy. ‘Run’ shows a perfect AUC of 1.00, indicating the model’s efficient performance in identifying running activity. The ‘Baseline’ activity, however, has a slightly lower AUC of 0.94, suggesting that the model is still performing well. Based on our analysis, we still conclude that there is some room for improvement in differentiating ‘Baseline’ from other activities.

### 4.4 Experiment 4: resource and time efficiency analysis of proposed system

In our fourth experiment, our focus is on evaluating the resource utilization and time efficiency of the proposed system. This analysis is most important for understanding the system’s overall performance, practicality, and feasibility in real-world applications for smart and personalized healthcare management, specifically where the timely and efficient processing of data can be of utmost importance In the case of the mHealth dataset, the system demonstrated a runtime of 7,632 s and memory usage of 4,421 MB. These metrics are indicative of the computational demands required for processing health-related data within this dataset. The runtime reflects the system’s ability to recognize different activities in a reasonable time frame. Other than time-based efficiency, the system must be memory efficient as well, or at least within an acceptable range for modern computational systems, ensuring that the system does not impose excessive demands on hardware resources. Similarly, for the ScientISST MOVE dataset, the proposed system reported a runtime of 4,632 s and consumed 3,611 MB of memory. With minimum computational time, in comparison to the mHealth dataset, suggests a more efficient processing capability, possibly due to variations in dataset complexity or size. The memory footprint is also lower, which could be attributed to more optimized data handling or a smaller feature set required for activity recognition in this dataset. Overall, our proposed system indicates an encouraging balance between resource consumption and time efficiency. These findings suggest that the system can be effectively deployed in environments where computational resources are limited. Due to better resource and time management, our proposed system is most feasible for mobile-based health applications, where the system’s efficiency can lead to longer battery life and more sustained monitoring capabilities. This indicates our proposed system will also perform well on different datasets, suggesting flexibility and robustness.

### 4.5 Experiment 5: comparisons with state of the art (SOTA)

**FIGURE 14:**
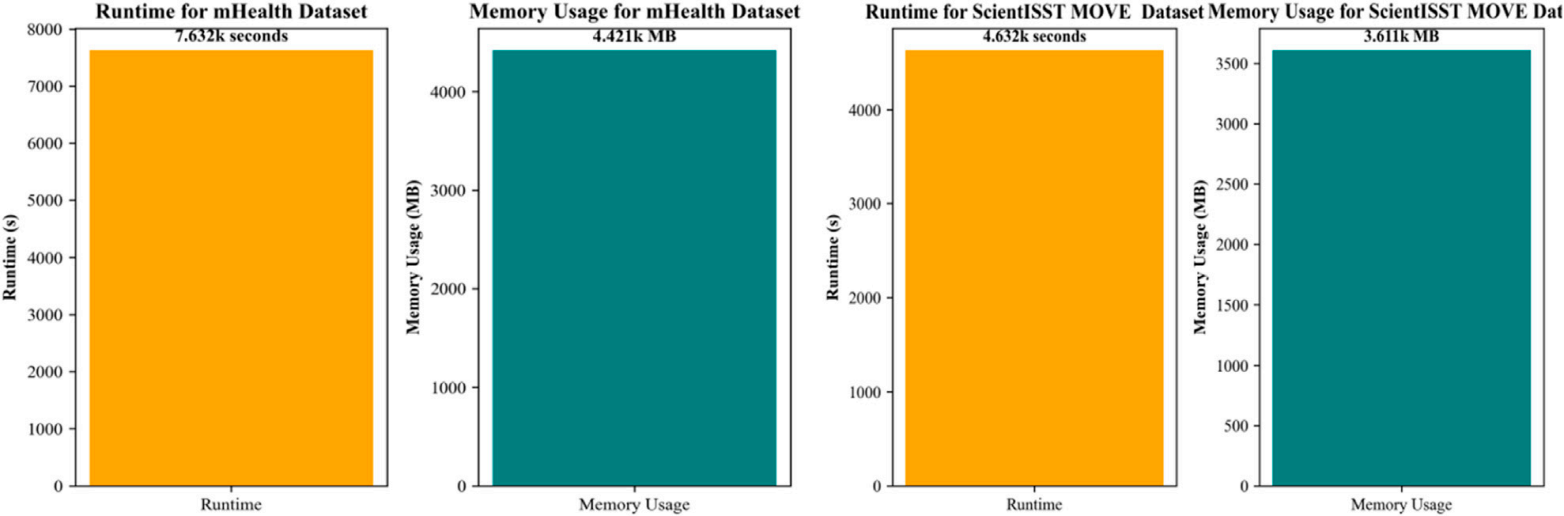
Runtime and Memory usage calculated over the ScientISST MOVE and mHealth Dataset.

In Kutlay and Gagula-Palalic (2015), the authors employed Multilayer Perceptron (MLP) and Support Vector Machine (SVM) algorithms to analyze the mHealth dataset, achieving an accuracy of 91% with the MLP model. However, our system not only achieves a higher accuracy (94.67%) but also shows superior performance in terms of precision and recall, particularly for dynamic activities such as “Running” and “Jump front and back,” where our model reached perfect scores in terms of both precision and AUC (1.00) (Liu C. et al., 2021; Zhang and Jiang, 2021), federated learning was implemented as a privacy-centric approach, achieving 90% accuracy on the WESAD dataset. While the focus of that study was on privacy, our study emphasizes a comprehensive evaluation of performance across multiple parameters. In particular, we highlight our model’s F1 scores and recall, which are critical in ensuring that key activities are detected reliably, especially in healthcare applications. In a comparative study by (Halloran and Curry, 2019), XGBoost achieved an accuracy of 89.97% on the mHealth dataset. Our proposed system overtakes this, with an accuracy of 94.67% and a higher AUC for key activities. Additionally, our system provides a better balance between precision and recall across a broader range of activities, offering significant improvements in the classification of both high-energy and low-energy tasks. Resource and efficiency plots are presented in Figure 14 (See Table 4).

**TABLE 4:** Comparisons with state of the art.

Method	Accuracy %	
	mHealth	ScientISST MOVE
Kutlay and Gagula-Palalic (2015)	91.70	—
Liu, (2021)	90.20	—
Halloran and Curry (2019)	89.97	—
Proposed	**94.67**	**95.12**

The bold values represents our proposed system accuracy.

## 5 Implication of the proposed system

Based on ROC, precision, recall, and F-1 score, it is evident that our proposed system is robust and applicable in emergency response, healthcare monitoring and fitness tracking, and many more where accuracy and precision matter a lot. Our findings are based on our proposed system’s robust performance as indicated by the Receiver Operating Characteristic (ROC) curves and the precision, recall, and F1 score metrics. The ROC curves for both the mHealth and ScientISST MOVE datasets reveal high Area Under the Curve (AUC) values for a majority of activities. Such high AUC values denote not only the system’s ability to correctly classify activities with high true positive rates but also its proficiency in maintaining low false positive rates across various thresholds. In the case of AUCs close to 1, such as ‘Running’ and ‘Jump front and back’ in the case of the mHealth dataset, and ‘Run’ in the ScientISST MOVE dataset, the implications are particularly encouraging for applications that demand a high degree of accuracy, like emergency response systems, where distinguishing between running and less vigorous activities could be critical. In the case of healthcare, precise detection of these activities can contribute to the development of smart patient monitoring systems, allowing for accurate assessment of patient mobility and activity levels, which are crucial for postoperative care and rehabilitation. The precision, recall, and F1 scores further underline the system’s reliability. High precision in activities like ‘Standing still’ and ‘Lying down’ suggests that the system could be successfully used in sedentary behavior research, where distinguishing between various low-energy activities is essential. On the other side, high recall in activities such as ‘Walking’ points to the system’s ability to capture these activities constantly, making it suitable for use in physical activity tracking and fitness apps, where missing an activity can lead to significant data inaccuracies. Moreover, the F1 scores, which reflect the balance between precision and recall, suggest that the system is highly capable of recognizing both static and dynamic activities. The system’s robust performance in dynamic and static activity recognition (Section 4) positions it for deployment in hospitals, remote monitoring, and elderly care. However, real-world adoption requires addressing regulatory (e.g., FDA certification), ethical (e.g., GDPR-compliant anonymization), and technical challenges (e.g., optimizing battery life via edge computing). Our future work will validate the system in clinical trials with variable sensor placements and integrate federated learning to enhance data privacy.

## 6 Conclusion and limitations

This research study proposed a comprehensive and advanced health monitoring system by integrating multimodal bio-signal from two well-known datasets, i.e., mHealth and ScientISST Move. Our proposed innovative healthcare monitoring system used an advanced AI technique, such as Deep Belief Network, which recognized and classified a wide range of physiological activities with enhanced accuracy and predictions. Moreover, advanced signal processing techniques such as Empirical Mode Decomposition (EMD) and Muscle Synergy Analysis, along with the use of filters for minimizing the impact of noise in the signal, have further enhanced the reliability and quality of the bio-signal data. Due to this modification, we have achieved higher precision, recall, and F1 score across all activities in both datasets. Our proposed integrated system is most suitable for real-world applications based on its efficiency, resource consumption, and time complexity, particularly in mobile-based deployments. Besides high accuracy, there are a few limitations as well, such as the deployment of sensor variability, motion artifacts from unpredictable movements, and complex patient conditions, like Parkinson’s tremors, may bring noise that is not available in our datasets, which have been used. Future research work is recommended to address its clinical trials across dense populations to validate its robustness under specific conditions.

## Data Availability

Publicly available datasets were analyzed in this study. This data can be found here: https://www.kaggle.com/datasets/gaurav2022/mobile-health; https://physionet.org/content/scientisst-move-biosignals/1.0.1/.

## References

[B1] Abdulmalek S. Nasir A. Jabbar W. A. Almuhaya M. A. M. Bairagi A. K. Khan M.A.-M. (2022). IoT-based healthcare-monitoring system towards improving quality of life: a review. Healthcare 10, 1993. 10.3390/healthcare10101993 36292441 PMC9601552

[B2] Agnuski M. Noga A. Surma M. Wójcik D. (2022). Modified triple-tuned bandpass filter with two concurrently tuned transmission zeros. Sensors 22, 9760. 10.3390/s22249760 36560128 PMC9787736

[B3] Ahmed S. Irfan S. Kiran N. Masood N. Anjum N. Ramzan N. (2023). Remote health monitoring systems for elderly people: a survey. Sensors 23, 7095. 10.3390/s23167095 37631632 PMC10458487

[B4] Alam R.-u. Zhao H. Goodwin A. Kavehei O. McEwan A. (2020). Differences in power spectral densities and phase quanti-ties due to processing of EEG signals. Sensors 20, 6285. 10.3390/s20216285 33158213 PMC7662261

[B5] Alqarni M. A. (2021). RETRACTED ARTICLE: error-less data fusion for posture detection using smart healthcare systems and wearable sensors for patient monitoring. Pers. Ubiquit Comput. 28, 3. 10.1007/s00779-020-01518-9

[B6] Alsaify B. A. Almazari M. M. Alazrai R. Alouneh S. Daoud M. I. (2022). A CSI-based multi-environment human activity recognition framework. Appl. Sci. 12, 930. 10.3390/app12020930

[B7] Areias Saraiva J. Abreu M. Carmo A. S. Plácido da Silva H. Fred A. (2023). ScientISST MOVE: annotated wearable multi-modal biosignals recorded during everyday life activities in naturalistic environments. Version 1.0.0. physionet., 2023. 10.13026/sg89-qq52

[B8] Arquilla K. Webb A. K. Anderson A. P. (2022). Utility of the full ECG Waveform for stress classification. Sensors 22, 7034. 10.3390/s22187034 36146383 PMC9501111

[B9] Arshad M. H. Bilal M. Gani A. (2022). Human activity recognition: review, taxonomy and open challenges. Sensors 22, 6463. 10.3390/s22176463 36080922 PMC9460866

[B10] Banos O. Garcia R. Saez A. (2014). MHEALTH dataset. UCI Mach. Learn. Repos. 10.24432/C5TW22

[B11] Bobade P. Vani M. (2020). “Stress detection with machine learning and deep learning using multimodal physiological data,” in Proceedings of the 2020 second international conference on inventive research in computing applications (ICIRCA) (Coimbatore, India), 51–57. 10.1109/ICIRCA48905.2020.9183244

[B12] Cengiz A. B. Birant K. U. Cengiz M. Birant D. Baysari K. (2022). Improving the performance and explainability of indoor human activity recognition in the internet of things environment. Symmetry 14, 2022. 10.3390/sym14102022

[B13] Centeno-Bautista M. A. Rangel-Rodriguez A. H. Perez-Sanchez A. V. Amezquita-Sanchez J. P. Granados-Lieberman D. Valtierra-Rodriguez M. (2023). Electrocardiogram analysis by means of empirical mode decomposition-based methods and convolutional neural networks for sudden cardiac death detection. Appl. Sci. 13, 3569. 10.3390/app13063569

[B14] Chaddad A. Wu Y. Kateb R. Bouridane A. (2023). Electroencephalography signal processing: a comprehensive review and analysis of methods and techniques. Sensors 23, 6434. 10.3390/s23146434 37514728 PMC10385593

[B15] Delmastro F. Martino F. D. Dolciotti C. (2020). Cognitive training and stress detection in MCI frail older people through wearable sensors and machine learning. IEEE Access 8, 65573–65590. 10.1109/ACCESS.2020.2985301

[B16] Demrozi F. Bacchin R. Tamburin S. Cristani M. Pravadelli G. (2020). Toward a wearable system for predicting freezing of gait in people affected by Parkinson's disease. IEEE J. Biomed. Health Inf. 24, 2444–2451. 10.1109/JBHI.2019.2952618 31715577

[B17] Díaz Á. Kaschel H. (2023). Scalable electronic health record management system using a dual-channel blockchain hy-per ledger fabric. Systems 11, 346. 10.3390/systems11070346

[B18] Ding X. Li Z. Yu J. Xie W. Li X. Jiang T. (2023). A novel lightweight human activity recognition method via L-CTCN. Sensors 23, 9681. 10.3390/s23249681 38139528 PMC10747825

[B19] Du Y. Lim Y. Tan Y. (2019). A novel human activity recognition and prediction in smart home based on interaction. Sensors (Basel). 19, 4474. 10.3390/s19204474 31619005 PMC6833365

[B20] Gao Z. Liu D. Huang K. Huang Y. (2019). Context-aware human activity and smartphone position-mining with motion sensors. Remote Sens. 11, 2531. 10.3390/rs11212531

[B21] Goecks J. Jalili V. Heiser L. M. Gray J. W. (2020). How machine learning will transform biomedicine. Cell 181, 92–101. 10.1016/j.cell.2020.03.022 32243801 PMC7141410

[B22] Guo S. Qi J. Wang Y. Liu Z. Li J. (2025). A flexible impact sensor of interpenetrating-phase composite architecture with high mechanical stability and energy-absorbing capability. Adv. Funct. Mater., 2419882. 10.1002/adfm.202419882

[B23] Halloran J. O. Curry E. (2019). A comparison of deep learning models in human activity recognition and behavioural pre-diction on the MHEALTH dataset. AICS 2563, 212–223.

[B24] Han E.-G. Kang T.-K. Lim M.-T. (2023). Physiological signal-based real-time emotion recognition based on exploiting mutual information with physiologically common features. Electronics 12, 2933. 10.3390/electronics12132933

[B25] Hayano J. Yamamoto H. Nonaka I. Komazawa M. Itao K. Ueda N. (2020). Quantitative detection of sleep apnea with wearable watch device. PLoS ONE 15 (11), e0237279. 10.1371/journal.pone.0237279 33166293 PMC7652322

[B26] Hu J. Jiang H. Xiao Z. Chen S. Dustdar S. Liu J. (2024). HeadTrack: real-time human–computer interaction via wireless earphones. IEEE J. Sel. Areas Commun. 42 (4), 990–1002. 10.1109/JSAC.2023.3345381

[B27] Hu L. Zhao K. Zhou X. Ling B.W.-K. Liao G. (2020). Empirical mode decomposition based multi-modal activity recognition. Sensors 20, 6055. 10.3390/s20216055 33114352 PMC7662633

[B28] Hu Z. Ren L. Wei G. Qian Z. Liang W. Chen W. (2023). Energy flow and functional behavior of individual muscles at different speeds during human walking. IEEE Trans. Neural Syst. Rehabil. Eng. 31, 294–303. 10.1109/TNSRE.2022.3221986 36374868

[B29] Hussian A. Mateen A. Amin F. Abid M. A. Ullah S. (2023). Health monitoring apps: an evaluation of the persuasive system design model for human wellbeing. Information 14, 412. 10.3390/info14070412

[B30] Islam M. R. Kabir M. M. Mridha M. F. Alfarhood S. Safran M. Che D. (2023). Deep learning-based IoT system for remote monitoring and early detection of health issues in real-time. Sensors 23, 5204. 10.3390/s23115204 37299933 PMC10255698

[B31] Jabeen T. Jabeen I. Ashraf H. Ullah A. Jhanjhi N. Z. Ghoniem R. M. (2023). Smart wireless sensor technology for healthcare monitoring system using cognitive radio networks. Sensors 23, 6104. 10.3390/s23136104 37447952 PMC10346715

[B32] Juan J. Greiner R. (2021). An introduction to machine learning approaches for biomedical research. Front. Med. 8, 771607. 10.3389/fmed.2021.771607 PMC8716730 34977072

[B33] Junaid S. B. Imam A. A. Balogun A. O. De Silva L. C. Surakat Y. A. Kumar G. (2022). Recent advancements in emerging technologies for healthcare management systems: a survey. Healthcare 10, 1940. 10.3390/healthcare10101940 36292387 PMC9601636

[B34] Khan M. A. Din I. U. Kim B.-S. Almogren A. (2023). Visualization of remote patient monitoring system based on internet of medical things. Sustainability 15, 8120. 10.3390/su15108120

[B35] Ku A. Rahim K. N. Elamvazuthi I. Izhar L. I. Capi G. (2018). Classification of human daily activities using ensemble methods based on smartphone inertial sensors. Sensors 18, 4132. 10.3390/s18124132 30486242 PMC6308488

[B36] Kumar A. Chakravarthy S. Nanthaamornphong A. (2023). Energy-efficient deep neural networks for EEG signal noise Re-duction in next-generation green wireless networks and industrial IoT applications. Symmetry 15, 2129. 10.3390/sym15122129

[B37] Kutlay M. A. Gagula-Palalic S. (2015). Application of machine learning in healthcare: analysis on MHEALTH dataset. Southeast europe J. Soft Comput. 4, 46–51.

[B38] Li C. Bian Y. Zhao Z. Liu Y. Guo Y. (2024). Advances in BiointegratedWearable and ImplantableOptoelectronic devices for CardiacHealthcare. Cyborg Bionic Syst.2024 5, 0172. 10.34133/cbsystems.0172 PMC11486891 39431246

[B39] Liang X. Huang Z. Yang S. Qiu L. (2018). Device-free motion and trajectory detection via RFID. ACM Trans. Embed. Comput. Syst. 17, 1–27. 10.1145/3230644

[B40] Liu A. Zhai Y. Xu N. Nie W. Li W. Zhang Y. (2022). Region-aware image captioning via interaction learning. IEEE Trans. Circuits Syst. Video Technol. 32, 3685–3696. 10.1109/TCSVT.2021.3107035

[B41] Liu C. Wu T. Li Z. Ma T. Huang J. (2022). Robust online tensor completion for IoT streaming data recovery. IEEE Trans. Neural Netw. Learn. Syst. 34, 10178–10192. 10.1109/TNNLS.2022.3165076 35436201

[B42] Liu H. (2021). Biosignal processing and activity modeling for multimodal human activity recognition. 10.26092/elib/1219

[B43] Liu J. Goetz S. Sen A. Tewari A. (2021). Learning from others without sacrificing privacy: simulation comparing centralized and federated machine learning on mobile health data. JMIR Mhealth Uhealth 9, e23728. 10.2196/23728 33783362 PMC8044739

[B44] Ma X. Dong Z. Quan W. Dong Y. Tan Y. (2022). Real-time assessment of asphalt pavement moduli and traffic loads using monitoring data from built-in sensors: optimal sensor placement and identification algorithm. Mech. Syst. Signal Process. 187, 109930. 10.1016/j.ymssp.2022.109930

[B45] Malik S. A. Parah S. A. Aljuaid H. Malik B. A. (2023). An iterative filtering based ECG denoising using lifting wavelet transform technique. Electronics 12, 387. 10.3390/electronics12020387

[B46] Meisel C. El Atrache R. Jackson M. Schubach S. Ufongene C. Loddenkemper T. (2020). Machine learning from wristband sensor data for wearable, noninvasive seizure forecasting. Noninvasive Seizure Forecast. Epilepsia. 61, 2653–2666. 10.1111/epi.16719 33040327

[B47] Mishra A. Bhusnur S. (2022). “A new adaptive modeling and denoising of real ECG signal,” in Proceedings of the 2022 IEEE 3rd global conference for advancement in technology (GCAT) (Bangalore, India), 1–6. 10.1109/GCAT55367.2022.9971940

[B48] Murad A. Pyun J.-Y. (2017). Deep recurrent neural networks for human activity recognition. Sensors 17, 2556. 10.3390/s17112556 29113103 PMC5712979

[B49] Ogbuabor G. La R. (2018). “Human activity recognition for healthcare using smartphones,” in Proceedings of the 2018 10th international conference on machine learning and computing (ICMLC '18) (New York, NY, USA), 41–46. 10.1145/3195106.3195157

[B50] Paraschiakos S. Cachucho R. Moed M. van Heemst D. Mooijaart S. Slagboom E. P. (2020). Activity recognition using wearable sensors for tracking the elderly. User Model User-Adap Inter 30, 567–605. 10.1007/s11257-020-09268-2

[B51] Pires I. M. Marques G. Garcia N. M. Pombo N. Flórez-Revuelta F. Spinsante S. (2019). Recognition of activities of daily living and environments using acoustic sensors embedded on mobile devices. Electronics 8, 1499. 10.3390/electronics8121499

[B52] Qi M. Cui S. Chang X. Xu Y. Meng H. Wang Y. (2022). Multi-region nonuniform brightness correction algorithm based on L-channel gamma transform. Secur. Commun. Netw. 2022, 1–9. 10.1155/2022/2675950

[B53] Qu J. Mao B. Li Z. Xu Y. Zhou K. Cao X. (2023). Recent progress in advanced tactile sensing technologies for soft grippers. Adv. Funct. Mater. 33, 2306249. 10.1002/adfm.202306249

[B54] Qu J. Yuan Q. Li Z. Wang Z. Xu F. Fan Q. (2023). All-in-one strain-triboelectric sensors based on environment-friendly ionic hydrogel for wearable sensing and underwater soft robotic grasping. Nano Energy 111, 108387. 10.1016/j.nanoen.2023.108387

[B55] Rastogi N. Mehra R. (2013). Analysis of Butterworth and Chebyshev filters for ECG denoising using wavelets. IOSR J. Electron. Commun. Eng. 6, 37–44.

[B56] Riedel D. E. Venkatesh S. Liu W. (2008). Recognising online spatial activities using a bioinformatics inspired sequence alignment approach. Pattern Recognit. 41, 3481–3492. 10.1016/j.patcog.2008.04.019

[B57] Rodrigues J. Liu H. Folgado D. Belo D. Schultz T. Gamboa H. (2022). Feature-based information retrieval of multimodal biosignals with a self-similarity matrix: focus on automatic segmentation. Biosensors 12, 1182. 10.3390/bios12121182 36551149 PMC9776348

[B58] Sei-ichi S. Yonatan H. Dai O. Mitsuhiro H. (2023). Ground reaction force and moment estimation through EMG sensing using long short-term memory network during posture coordination. Cyborg Bionic Syst. 4, 0016. 10.34133/cbsystems.0016 37000191 PMC10044327

[B59] Singh R. Kushwaha A. K. S. Srivastava R. (2023). Recent trends in human activity recognition – a comparative study. Cogn. Syst. Res. 77, 30–44. 10.1016/j.cogsys.2022.10.003

[B60] Strzelecki M. Badura P. (2022). Machine learning for biomedical application. Appl. Sci. 12, 2022. 10.3390/app12042022

[B61] Su S. Zhu Z. Wan S. Sheng F. Xiong T. Shen S. (2023). An ECG signal acquisition and analysis system based on machine learning with model fusion. Sensors 23, 7643. 10.3390/s23177643 37688099 PMC10490810

[B62] Tao He Yabin Z. Xu L. Jiamin Li Longyang L. Wenfeng Z. (2023). A highly energy-efficient body-coupled transceiver employing a power-on-demand amplifier. Cyborg Bionic Syst. 4, 0030. 10.34133/cbsystems.0030 37559940 PMC10408381

[B63] Wang F. Wang H. Zhou X. Fu R. (2022). A driving fatigue feature detection method based on multifractal theory. IEEE Sensors J. 22, 19046–19059. 10.1109/JSEN.2022.3201015

[B64] Wang L. Song F. Zhou T. H. Hao J. Ryu K. H. (2023). EEG and ECG-based multi-sensor fusion computing for real-time fatigue driving recognition based on feedback mechanism. Sensors 23, 8386. 10.3390/s23208386 37896480 PMC10611368

[B65] Wang X. Shang J. (2023). Human activity recognition based on two-channel residual–GRU–ECA module with two types of sensors. Electronics 12, 1622. 10.3390/electronics12071622

[B66] Wen C. Huang Y. Zheng L. Liu W. Davidson T. N. (2023). Transmit Waveform design for dual-function Ra-dar-Communication systems via hybrid linear-nonlinear precoding. IEEE Trans. Signal Process. 71, 2130–2145. 10.1109/TSP.2023.3278858

[B67] Xing Y. Yang K. Lu A. Mackie K. Guo F. (2024). Sensors and DevicesGuided by artificial intelligencefor personalized pain medicine. Cyborg Bionic Syst. 5, 0160. 10.34133/cbsystems.0160 39282019 PMC11395709

[B68] Yan L. Shi Y. Wei M. Wu Y. (2023). Multi-feature fusing local directional ternary pattern for facial expressions signal recognition based on video communication system. Alex. Eng. J. 63, 307–320. 10.1016/j.aej.2022.08.003

[B69] Yao Y. Shu F. Li Z. Cheng X. Wu L. (2023). Secure transmission scheme based on joint radar and communication in mobile vehicular networks. IEEE Trans. Intell. Transp. Syst. 24, 10027–10037. 10.1109/TITS.2023.3271452

[B70] Yin Y. Guo C. Mu Q. Li W. Yang H. He Y. (2024). Dual-sensing nano-yarns for real-time pH and temperature monitoring in smart textiles. Chem. Eng. J. 500, 157115. 10.1016/j.cej.2024.157115

[B71] Zhang S. Li Y. Zhang S. Shahabi F. Xia S. Deng Y. (2022). Deep learning in human activity recognition with wearable sensors: a review on advances. Sensors 22, 1476. 10.3390/s22041476 35214377 PMC8879042

[B72] Zhang X. Huang D. Li H. Zhang Y. Xia Y. Liu J. (2023). Self-training maximum classifier discrepancy for EEG emotion recognition. CAAI Trans. Intell. Technol. 8, 1480–1491. 10.1049/cit2.12174

[B73] Zhang X. Jiang S. (2021). “Application of fourier transform and Butterworth filter in signal denoising,” in Proceedings of the 2021 6th international conference on intelligent computing and signal processing (ICSP) (Xi'an, China), 1277–1281. 10.1109/ICSP51882.2021.9408933

[B74] Zhang X. Liu Q. He D. Suo H. Zhao C. (2023). Electrocardiogram-based biometric identification using mixed feature ex-traction and sparse representation. Sensors 23, 9179. 10.3390/s23229179 38005564 PMC10675745

[B75] Zhao Y. Li B. Zhong M. Fan H. Li Z. Lyu S. (2025). Highly sensitive, wearable piezoresistive methylcellulose/chitosan@MXene aerogel sensor array for real-time monitoring of physiological signals of pilots. Sci. China Mater. 68 (2), 542–551. 10.1007/s40843-024-3188-4

[B76] Zhao Z. Xu G. Zhang N. Zhang Q. (2022). Performance analysis of the hybrid satellite-terrestrial relay network with opportunistic scheduling over generalized fading channels. IEEE Trans. Veh. Technol. 71, 2914–2924. 10.1109/TVT.2021.3139885

[B77] Zheng Y. Lv X. Qian L. Liu X. (2022). An optimal BP neural network track prediction method based on a GA–ACO hybrid algorithm. J. Mar. Sci. Eng. 10, 1399. 10.3390/jmse10101399

[B78] Zhou X. Zhang L. (2022). SA-FPN: an effective feature pyramid network for crowded human detection. Appl. Intell. 52, 12556–12568. 10.1007/s10489-021-03121-8

[B79] Zhu T. Ding H. Wang C. Liu Y. Xiao S. Yang G. (2023). Parameters calibration of the GISSMO failure model for SUS301l-MT. Chin. J. Mech. Eng. 36, 20. 10.1186/s10033-023-00844-2

[B80] Zyout A. Alquran H. Mustafa W. A. Alqudah A. M. (2023). Advanced time-frequency methods for ECG waves recognition. Diagnostics 13, 308. 10.3390/diagnostics13020308 36673118 PMC9858079

